# Carpometacarpal dislocation of the thumb associated with fracture of the trapezium

**DOI:** 10.1007/s10195-014-0288-9

**Published:** 2014-03-27

**Authors:** Ozkan Kose, Mert Keskinbora, Ferhat Guler

**Affiliations:** 1 Department of Orthopaedics and Traumatology, Antalya Education and Research Hospital, Kultur mah. 3025 sk. Durukent Sit. F Blok Daire 22, Kepez, Antalya, Turkey; 2 Faculty of Medicine, Department of Orthoapedics and Traumatology, Medipol University, Istanbul, Turkey

**Keywords:** CMC dislocation, Trapezium fracture, Trapeziometacarpal fracture–dislocation

## Abstract

Carpometacarpal dislocation (CMC) of the thumb associated with fracture of trapezium is an extremely rare injury, with only 12 cases that sustained similar injuries reported in the literature. In this article, another patient with this rare injury was reported, and all previously published cases were extensively reviewed. The presented case and all previously published cases had a longitudinally oriented trapezium fracture, which is naturally unstable and almost always associated with dislocation of the CMC joint. In contrast to previous descriptions, we believe that CMC joint dislocation and trapezium fracture are not two distinct pathologies that occur simultaneously by chance but share cause and consequence.

## Introduction

Pure carpometacarpal (CMC) dislocations of the thumb are rare injuries that account for <1 % of all hand injuries [1]. However, these injuries frequently occur in conjunction with avulsion of the metacarpal base due to thick and strong volar ligamentous attachments, the so-called Bennett’s fracture–dislocation [1–4]. On the other hand, CMC dislocation of the thumb associated with fracture of trapezium is an extremely rare injury. To the best of our knowledge, only a few cases of the combination of such injuries are reported in English literature (Table 1) [5–15]. The purpose of this report is to describe a patient with CMC dislocation of the thumb associated with fracture of trapezium and to discuss anatomy, mechanism of injury, treatment options, and outcomes in light of current literature.

**Table 1:** Previously published cases in the English literature of carpometacarpal (CMC) dislocation of the thumb associated with trapezium fracture

References	Sex	Age (years)	Mechanism of injury	Trapezium fracture classification	Treatment	Follow-up (months)	Result
Tolat and Jones [7]	Male	14	Fall onto an outstretched hand (skateboard)	IIa	Closed reduction splinting 6 weeks	2	Excellent without instability, full ROM
Mody and Dias [14]	Male	24	Motorbike accident	IIa	Open reduction and K-wire fixation; ligament reconstruction	6	Excellent
Kukreti and Harrington [13]	Not reported	26	Sport injury (rugby tackling)	IIa	Closed reduction and K-wire fixation	12	Slight pain, minimal loss of CMC flexion of CMC
Garavaglia et al. [11]	Female	20	Fall while holding the handle of a bucket	IIa	Open reduction and screw fixation	12	Excellent
Garneti and Tuson [9]	Male	24	Sport injury (rugby tackling)	IV	Open reduction and internal fixation with a minifragment 2.7-mm lag compression screw	12	Excellent
	Male	18	Sport injury (rugby)	IV	Open reduction and internal fixation with a single 2.7-mm lag screw	9	Excellent
Afshar and Mirzatoloei [6]	Male	30	Motorbike accident	IIa	Closed reduction and K-wire fixation	Not reported	No pain and instability, full ROM
Parker et al. [10]	Male	12	Fall onto an outstretched hand (rollerblade)	IIa	Closed reduction, spanning external fixation	36	Excellent
Morizaki and Miura [15]	Male	31	Fall onto flexed thumb	IIa	Open reduction and internal fixation using suture anchor and K-wire fixation	12	Excellent
Chamseddine et al. [5]	Male	23	Road accident	IV	Open reduction and K-wire fixation	9	Excellent
Ramoutar et al. [12]	Male	27	Fall onto an outstretched hand (football)	IIa	Closed reduction and K-wire fixation	6	Excellent
Mumtaz and Drabu [8]	Male	14	Direct trauma due to hammer hit (open injury)	IV	Irrigation, debridement, and K-wire fixation	12	Gross impairment in opposition and abduction
This case	Male	32	Motorbike accident	IIa	Closed reduction and splinting for 6 weeks	6	Excellent

*ROM* range of motion

## Case report

**Fig. 1:**
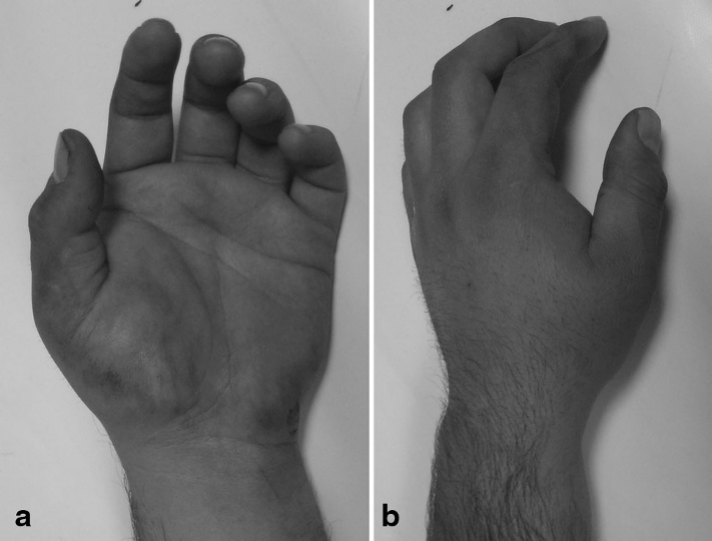
Volar (**a**) and dorsal (**b**) appearance at presentation

**Fig. 2:**
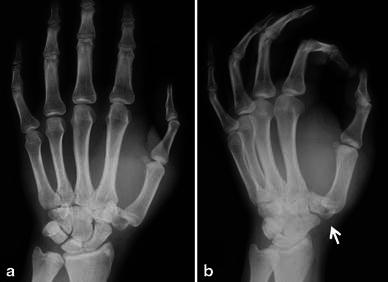
Anteroposterior (**a**) and oblique (**b**) radiographs. *White arrow* trapezium fracture

A 26-year-old electrician was involved in a motorcycle accident and brought to our emergency department. He complained of pain, swelling, and functional impairment of his left thumb. On physical examination, there was edema and tenderness over the thenar area (Fig. 1). Dorsoradial subcutaneous prominence was palpated at the base of right thumb. Thumb movements were restricted and painful in all directions. Neurovascular examination was normal. Anteroposterior and oblique hand radiographs revealed CMC dislocation of the thumb associated with trapezium fracture (Fig. 2).

**Fig. 3:**
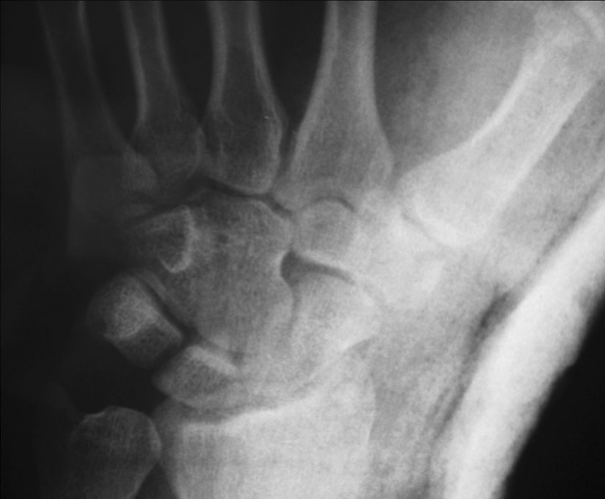
Postreduction radiograph of the carpometacarpal (CMC) joint showing adequate reduction and a congruent joint

**Fig. 4:**
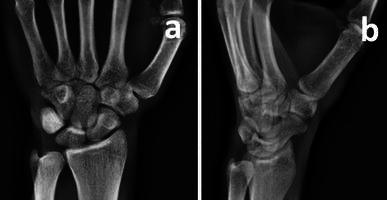
Final hand radiographs 6 months after injury

With the patient under conscious sedation, closed reduction was achieved by applying distraction to the thumb, followed by volarly directed pressure over the base of the thumb metacarpal. During reduction, a palpable click was felt, and the dorsoradial bony prominence disappeared. The thumb was immobilized in a plaster cast, and postreduction radiographs confirmed concentric relocation of the joint and the fracture without a significant step in the articular surface (Fig. 3). Immobilization continued for 6 weeks, and the patient was referred for physiotherapy. After cast removal, passive and active range of motion (ROM) exercises were started immediately and encouraged. To strengthen thumb grip and pinch, the patient was advised to clench a sponge ball as many times as possible. At the final follow-up 6 months after initial injury, the patient had gained full ROM in the CMC joint, without pain or instability. Final hand radiographs displayed a congruent CMC joint and trapezium fracture union (Fig. 4).

## Discussion

Although the thumb CMC joint has wide ROM, which goes from extension through abduction to flexion, it is a highly stable joint. Integrity between mobility and stability is essential for performing an effective key pinch and grasp [5]. Thumb CMC joint stability is provided by several structures, including the joint capsule, dorsal and volar ligaments, tendons transpassing the joint, and the saddle-shaped trapeziometacarpal (TMP) joint configuration [3, 4]. Four main ligamentous structures are accepted to be the primary source of static stability: anterior oblique ligament (AOL), intermetacarpal ligament, radial collateral ligament (RCL), and palmar oblique ligament [3]. Several biomechanical and cadaver studies investigating the contribution of these ligaments to thumb stability and preventing dorsoradial dislocation of the TMP joint showed that RCL is the primary restraint against dorsal dislocation [1–4].

Two different mechanism of injury have been proposed for CMC dislocation and associated fractures of the trapezium [1, 2, 16]. According to the first mechanism of injury, CMC dislocation of the thumb occurs from axial loading on a flexed thumb metacarpal, which drives the metacarpal base dorsally over the trapezium and ruptures the RCL. TCL rupture results in dorsal dislocation, which may be a pure dislocation without any accompanying fracture [2]. In pure CMC dislocations, the AOL is also torn or stripped subperiosteally. During this injury, if AOL avulses a piece of bone fragment from the base of the first metacarpal, Bennett’s fracture–dislocation occurs [6]. In some instances, a vertical split fracture of the trapezium may occur, with the pullout effect of the intact RCL and axial loading of the metacarpal base on the trapezium. In the second proposed mechanism of injury, a commissural shearing produced by the impact of an object against the first web space causes CMC joint dislocation. This type of injury may happen with a fall while grasping an object, or if the individual is thrown forward while holding the handlebar of a motorcycle. Varying impact angles result pure CMC dislocation, Bennett’s fracture–dislocation, or a trapezium fracture. If the vector of the force passes toward the trapezium, a trapezium fracture will occur [16]. Our patient was involved in a motorcycle accident and was thus more likely to be injured by commissural shearing forces produced by one of the handlebars during the collision. Other than these mechanisms of injuries, direct trauma (hit by a heavy hammer), fall onto an outstretched hand, and sporting injuries, have been reported in published cases [7–10].

**Fig. 5:**
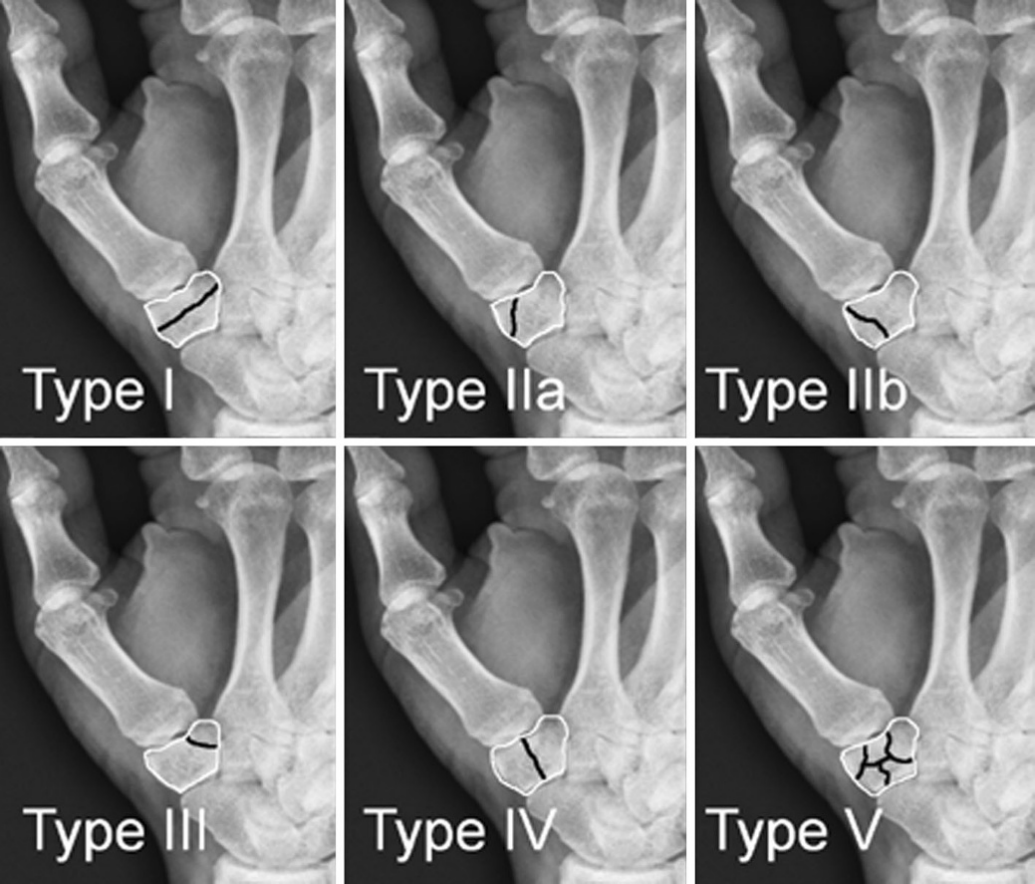
Classification of trapezium fractures by Walker et al. [16]. *Black lines* show the fracture

Trapezium fractures are rare injuries and comprise about 3 % of all carpal bone fractures [16]. In 1988, Walker et al. classified trapezium fractures into five different fracture patterns in a series of ten cases (Fig. 5). Most reported cases with a combination of thumb CMC dislocation and trapezium fracture presented as types IIa, IIb, or IV (longitudinally oriented trapezium fractures). Similarly, in the series presented by Walker et al., types IIb and IV were simultaneous thumb CMC dislocations. Therefore, it appears that types IIa, IIb, and IV fractures are almost always accompany by thumb CMC dislocation. Garavaglia et al. [11] described this injury as transtrapezium carpometacarpal dislocation of the thumb, which seems to be the appropriate nomenclature for this specific injury.

Isolated nondisplaced trapezium fractures may be missed on direct radiographs due to overlapping adjacent bones, particularly on anteroposterior hand radiographs. In addition to meticulous physical examination and standard anteroposterior and oblique hand X-rays; a true anteroposterior view of the CMC joint of the thumb (Robert’s view) with full pronation of the thumb is an effective imaging technique by which to visualize these fractures. In case of suspicion, CT imaging can be utilized for further detailed demonstration [12]. However, CMC dislocation associated with a trapezium fracture is usually evident, and there are only two cases which were initially missed the emergency department [11, 13]. Dorsoradial shift of the metacarpal, positive stress views and comparison with contralateral normal hand will be useful in confirming the subtle dislocations of the CMC joint. With an obvious dislocation of the thumb, attention must be paid for associated injuries including Bennett’s fracture-dislocation or trapezium fracture.

Several treatment methods are reported in the literature, ranging from closed reduction and cast immobilization to open reduction and ligamentous reconstruction, as summarized in Table 1. Usually, the dislocation can be reduced easily by thumb traction and abduction while gently pushing the metacarpal base medially [7, 13]. Nevertheless, the major factor affecting treatment outcome is reduction adequacy and maintenance. In this combination of injury, the RCL remains intact; therefore, if the joint is stable and reduction quality is good after closed manipulation, a thumb spica cast may be chosen [7]. Thumb extension and slight pronation in the cast allows approximation of the stripped AOL and may enable ligamentous healing while contributing the joint stability [10]. If conservative treatment is preferred, the patient should be checked for any radiological signs of reduction loss, particularly during the first 2 weeks after the injury. Serial radiographic follow-up is advocated to monitor the reduction quality achieved at initial reduction. Tolat et al. reported excellent outcome after conservative treatment in a skeletally immature patient (14 years old) [7]. Although, our patient was an adult, conservative treatment yielded an excellent outcome. However, closed reduction and percutanous pin fixation seems to be a more appropriate treatment method, as it is both minimally invasive and safe against loss of reduction during follow-up. We believe that extensive surgery, such as ligamentous reconstruction using tendon grafts, is overtreatment, because trapezium union and AOL healing provide adequate joint stability [6, 17].

As the follow-up period for reported cases is short (mean 11.1 months, range 2–36 months), it is difficult to comment on long-term consequences and prognosis, particularly the development of posttraumatic osteoarthritis. Theoretically, inadequate TMP joint reduction or trapezium malunion that leads to incongruency of the articular surface leads to osteoarthritis and loss of thumb function in the long term [16]. However, all reported cases expect one, a severe crush injury, resulted in good and excellent outcomes.

In conclusion thumb CMC dislocation associated with trapezium fracture is a rare injury, with few reported cases to date. Probable mechanism of injury is either axial loading on a flexed thumb or commissural shearing forces acting on the first web space. Radiographic evaluation of these patients should be done carefully to prevent missed diagnosis. Closed reduction and percutaneous K-wire fixation is sufficient to obtain a stable joint and promote proper ligamentous and bony healing. Although long-term follow-up is not available, prognosis is excellent in the short term.
